# Avermectin Derivatives, Pharmacokinetics, Therapeutic and Toxic Dosages, Mechanism of Action, and Their Biological Effects

**DOI:** 10.3390/ph13080196

**Published:** 2020-08-17

**Authors:** Gaber El-Saber Batiha, Ali Alqahtani, Omotayo B. Ilesanmi, Abdullah A. Saati, Amany El-Mleeh, Helal F. Hetta, Amany Magdy Beshbishy

**Affiliations:** 1 Department of Pharmacology and Therapeutics, Faculty of Veterinary Medicine, Damanhour University, Damanhour 22511, Egypt; 2 Department of Pharmacology, College of Pharmacy, King Khalid University, Guraiger, Abha 62529, Saudi Arabia; amsfr@kku.edu.sa; 3 Department of Biochemistry, Faculty of Science, Federal University Otuoke, Otuoke 561, Nigeria; ilesanmiob@fuotuoke.edu.ng; 4 Department of Community Medicine & Pilgrims Healthcare, Faculty of Medicine, Umm Al-Qura University Makkah, Mecca 24382, Saudi Arabia; aaasaati@uqu.edu.sa; 5 Department of Pharmacology, Faculty of Veterinary Medicine, Menoufia University, Shibin Al Kawm 32511, Egypt; Amany.ahmed1074@gmail.com; 6 Department of Medical Microbiology and Immunology, Faculty of Medicine, Assiut University, Assiut 71515, Egypt; hettahf@ucmail.uc.edu; 7 Department of Internal Medicine, University of Cincinnati College of Medicine, Cincinnati, OH 45221, USA; 8 National Research Center for Protozoan Diseases, Obihiro University of Agriculture and Veterinary Medicine, Nishi 2-13, Inada-cho, Obihiro 080-8555, Hokkaido, Japan

**Keywords:** avermectins, antihelminthic, insecticidal, pharmacokinetics, *Streptomyces avermitilis*

## Abstract

Avermectins are a group of drugs that occurs naturally as a product of fermenting *Streptomyces avermitilis*, an actinomycetes, isolated from the soil. Eight different structures, including ivermectin, abamectin, doramectin, eprinomectin, moxidectin, and selamectin, were isolated and divided into four major components (A1a, A2a, B1a and B2a) and four minor components (A1b, A2b, B1b, and B2b). Avermectins are generally used as a pesticide for the treatment of pests and parasitic worms as a result of their anthelmintic and insecticidal properties. Additionally, they possess anticancer, anti-diabetic, antiviral, antifungal, and are used for treatment of several metabolic disorders. Avermectin generally works by preventing the transmission of electrical impulse in the muscle and nerves of invertebrates, by amplifying the glutamate effects on the invertebrates-specific gated chloride channel. Avermectin has unwanted effects or reactions, especially when administered indiscriminately, which include respiratory failure, hypotension, and coma. The current review examines the mechanism of actions, biosynthesis, safety, pharmacokinetics, biological toxicity and activities of avermectins.

## 1. Introduction

Avermectins are a group of drugs with a multiple treatment target. They are generally used as a pesticide for the treatment of pests and parasitic worms as a result of their anthelmintic and insecticidal properties [1]. The compounds are derived from a 16-membered lactone ring. Avermectins occur naturally as a fermentation product of *Streptomyces avermitilis*, an actinomycetes, isolated from the soil. Eight different structures were isolated and divided into four major components (A1a, A2a, B1a and B2a) and four minor components (A1b, A2b, B1b, and B2b). Examples of avermectins include ivermectin, abamectin, doramectin, eprinomectin, moxidectin, and selamectin [2]. Their structures share similarity with antibacterial macrolides and antifungal macrocyclic polygenes but differ in the mechanism of action [3].

## 2. History

The first compound of avermectin was isolated in 1978. An actinomycete was isolated from a sample collected at Kawana, Ito City, and Shizuoka Prefecture, Japan. This organism was later sent to Merck Sharp and Dohme Research laboratories for analysis [4]. The isolated actinomycetes were cultured under a well-regulated condition and tested against *Nematospiroides dubius* in mice. The active compounds with antiparasitic effects have been isolated and identified as being a family of closely related compounds. The characterization of the antihelminthic compounds and the identification of the new species produced them were elucidated [5,6]. Yoko Takahishi and Co. have suggested renaming *Streptomyces avermitilis* as *Streptomyces avermectinius* [7].

## 3. Structure and Chemical Properties

Avermectin compounds share a similar structure with Milbemycin. The major difference between the two classes of compounds is at the position number 13 of the macrolide ring, where a bisoleandrosyloxy group is substituted. Other alterations include attachment of alkyl group of a different type at C-25 in both classes. Deleting the hydroxyl group at position number 12 results in the production of 13-deoxyavermectin (avermectin aglycone), which is very similar to some milbemycins and they can therefore be referred to as glycosylated milbemycins [8]. The bisoleandrosyl group at C-13 position is lipophilic and it is not important for the biological activity and is therefore often a target for chemical modification [9]. The easy accessibility to 4-position of avermectin has made it one of the most studied site for modification. Different chemical groups such as acyl, amino, or thio groups have been inserted at this position to alter the chemical properties of the drugs (solubility, stability distribution) without altering the potency of the parent drug [10,11]. Different derivatives of the parent drug have been produced by modifying the terminal sugar of avermectin to enhance the potency and biological activity, and improve its efficacy as an anthelmintic drug [5,6]. 24-hydroxymethyl-H_2_B_1a_ and 3′-*O*-desmethyl-H_2_B_1a_ are the two major metabolites of ivermectin that are found in the liver of cattle and swine. It was also observed that the concentrations of the metabolites were lower than the parent compounds. Also, the transformation of the drug in the soil produced a more polar by-product of ivermectin, which is less toxic than the parent drug to daphnids [12]. With respect to the physical and chemical properties of avermectin, they are less volatile, and poorly soluble in water, as 50% of ivermectin takes less than 6 h to be dissolved in water, while 90% of the drugs takes more than 16.8 days to be dissolved in water. They have a high vapor pressure, which makes it difficult for them to be distributed in the atmosphere [13]. Avermectins are generally soluble in organic solvents, such as ethanol, chloroform, diethyl ether, ethyl acetate. They have a high adsorption coefficient, which makes them less likely to accumulate in the water column. The hydrophobic properties (log *K*_ow_ = 3.2) and high-affinity organic matter make ivermectin to accumulate in the environment [9,14]. Experimental data obtained from field and laboratory research have shown that ivermectin residues are strongly attached to soil particles [15]. The high *K*_oc_ of ivermectin makes it immobile in the environment. Ivermectin is lipophilic, which may make it bio accumulate in animal tissues. However, the lipophilic nature of the drug is countered by the high molecular weight, which prevents it from crossing the biological membrane [10].

## 4. Avermectin Biosynthesis

**Figure 1:**
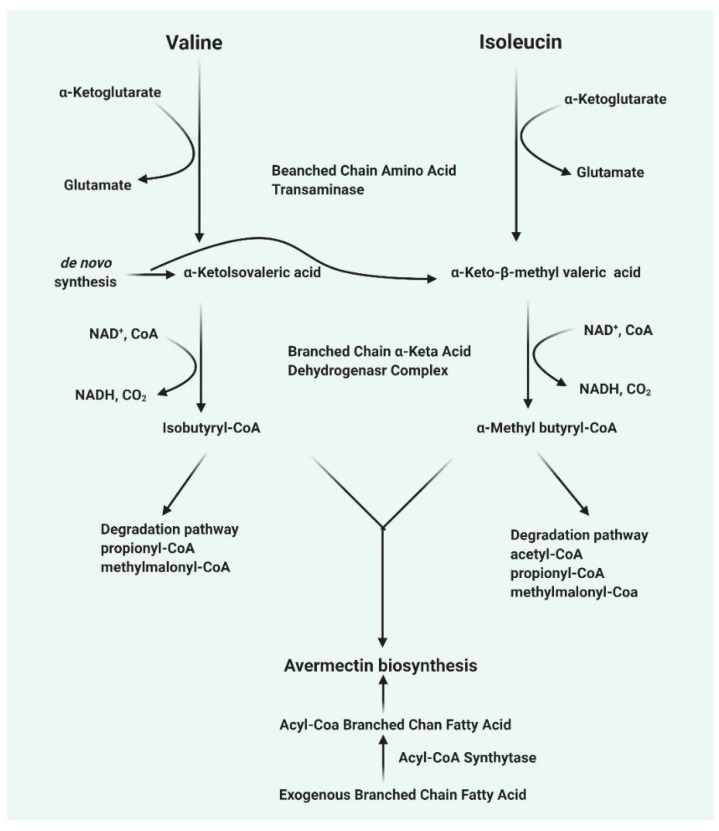
The biosynthesis of some classes of avermectins.

The mechanism of avermectin biosynthesis from *S. avermitilis* is present in a cluster of gene and has been sequenced. This cluster of genes encodes all the enzymes involved in various steps in the avermectin synthesis pathway [8]. The first step is the synthesis of avermectin aglycone catalyzed by polyketide synthases, followed by aglycone modification, synthesis of modified sugar and the avermectin aglycone is glycosylated with the modified sugar. Eight different types of avermectins are synthesized from these clusters of gene [16]. The polyketide synthase complex activity requires four proteins (AVES 1, AVES 2, AVES 3, and AVES 4) for the synthesis of the initial avermectin aglycone. There is a strong similarity between the activity of the enzyme and type I polyketide synthases [16]. The enzyme uses either isobutyrl CoA 2-methylbutyrl CoA as a substrate, after which seven acetate units and five propionate units are added to produce avermectin “A” or “B” series, respectively [16]. The first substrate is now removed from the enzyme complex (thioesterase domain of AVES4), via the cyclization to form cyclic esters. The other enzymatic activity involved the modification of the avermectin aglycone includes: (1) AveE which contains a Cyt P450 monooxygenase activity and catalyzes the cyclization between C6 and C8 to form a furan ring, (2) AveF which has a keto reductase activity that catalyzes the reduction of keto group on C5 to a hydroxyl group, using NAD(P)H as an electron donor, (3) The mechanism by which AveC acts as a dehydratase between C22–C23 in module 2 is not well understood, AveD possesses a methyltransferase activity that requires SAM [17,18]. Whether AveC or AveD acts on the aglycone determines whether the resulting avermectin aglycone produces avermectin series “A” or “B” and series 1 or 2, respectively. Downstream of AveA4 are none open reading frames (orf1, aveB-BVIII) that are involved in the synthesis of the sugar and glycosylation [17]. Analysis of orf1 sequence shows that it might probably possess a reductase activity that is active but might not be needed in the avermectin synthesis. AveBI is involved in avermectin aglycone glycosylation with an activated sugar (dTDP-sugar). AveBII-BVIII are involved in the synthesis of dTDP-L-oleandrose [19]. The summary of avermectin biosynthesis has been shown in Figure 1.

## 5. Avermectin Derivatives, Formulations, Therapeutic and Toxic Dosages, and Mechanism of Action

### 5.1. Avermectin Derivatives and Its Formulations

#### 5.1.1. Ivermectin

Ivermectin is the most common avermectin derivative. It is available in numerous forms and can be applied through different routes of administration. It is used by humans to treat gastrointestinal strongyloidiasis in the United States and other countries and has been used to treat onchocerciasis and strongyloidosis [10]. In animals, it is used to prevent heartworm, treat ectoparasite, and as a microfilaricide. Its dosage is dependent on the kind of treatment, i.e., 0.006 to 0.012 mg/kg has been used for heartworm prevention in dogs and 0.024 mg/kg in cats, 0.05 to 0.2 mg/kg as a macrofilaricide in dogs and 0.3 to 0.6 mg/kg to treat ectoparasites [20]. It is administered at high concentration in animals (10–18.7 mg/mL) that may lead to overdose due to wrong calculation or exposure [10]. Exposure may also be from the remnants of the de-wormer or drippings or droplets of the horse’s mouth when dewormed, or from the excreta of treated animals. For example, a study showed that the peak amount of ivermectin was 2.5 mg/kg of horse excreta after 2.5 days post-exposure [21]. This meant that for a collie to be exposed to a mild toxic level of ivermectin (0.1 mg/kg), a 27.3 kg (60 lb) collie homozygous for *ABCB1-1*Δ would ingest about 1.1 kg (2.4lb) of excreta [22]. Some breeds of dogs have been shown to develop clinical symptoms following ivermectin ingestion. However, it was not known whether dogs have defects in the *ABCB1* gene, particularly when the dose was between 0.08–0.34 mg/kg [23]. It was also shown that dose higher than 0.2 mg/kg resulted in mild symptoms in normal animals and developed severe signs when the dose was above 1 mg/kg [23,24]. The study also found that German shepherds are sensitive to low doses of ivermectin, with a small percentage having a defect in *ABCB1* gene, which could be correlated with clinical signs observed in normal dogs at low doses [25]. However, these problems have not been encountered with therapeutic doses to avoid heartworm in normal or *ABCB1* gene defective dogs. No clinical signs were observed when collies sensitive to ivermectin administered at a high dose of 0.06 mg/kg [26]. Patients with *ABCB1* gene defect can develop clinical signs when administered a high dose of ivermectin as a microfilaricide or for demodicosis. Sometimes dogs with normal *ABCB1* genotype can develop clinical signs when administered a high dose of ivermectin. Other clinical symptoms have been observed including bradycardia, tremors, hypersalivation, lethargy, ataxia, and blindness [26,27]. Several derivatives were developed including eprinomectin, which shows prolonged activity and no milk withdrawal and selamectin with a greater margin of safety than IVM in MDR1 mutated dogs [28].

#### 5.1.2. Moxidectin

Moxidectin is an example of avermectin that is used in horses and ruminants at high concentrations to prevent heartworms. It is administered through different routes such as topical, subcutaneous, oral route. A study documented that dogs can be exposed to moxidectin through horse dungs, with as much as 2.6 mg/kg of moxidectin measured after 2.5 days treatment [21]. Moxidectin is also administered to cats to prevent heartworm [20]. Some of the adverse effects of moxidectin administration in dogs include bradycardia, blindness, hypersalivation, hyperthermia, ataxia, respiratory depression, and coma [29].

#### 5.1.3. Selamectin

Selamectin is another type of avermectin that is used topically for preventing and killing heartworm and ear mites in cats and dogs, respectively [30]. Other treatment includes sarcoptic mange and tick infestation in dogs and ascarids and hookworms in cats. Adverse reactions were reported following oral exposure to selamectin, including vomiting, drooling, retching, licking of lips, lethargy, agitation, anorexia, and ataxia [31].

#### 5.1.4. Abamectin (Avermectin B_1_)

Abamectin, also known as avermectin B1 is used to regulate insects such as mites and cockroaches. They are often used with a bait to attract insects and sprayed at a concentration between 0.15% to 2%, while sprayed in a concentration between 0.01% and 0.05% for cockroach [32]. Their low concentration makes it rare to observe signs and symptoms. Some experiments involving sub-chronic toxicity of abamectin in rats, dogs, and mice have shown that it is slightly more toxic than ivermectin. Some of the clinical symptoms in dogs exposed to abamectin includes vomiting, ataxia, hypersalivation, lethargy, mydriasis, diarrhea, and gastrointestinal disturbance [33].

#### 5.1.5. Milbemycin

Milbemycin is another type of avermectin that is also used to prevent heartworms in dogs and cats and in a 0.1% otic solution for the treatment of ear mites [22]. It is administered orally in a chewable form. The low concentration makes it difficult for overdose. It is administered at a dose of 0.5 mg/kg and 2 mg/kg to dogs and cats, respectively to prevent heartworm. Dogs sensitive to ivermectin have been shown to develop mild symptoms including lethargy, hypersalivation, ataxia, and mydriasis when administered at a high dose ranging between 5 to 10 mg/kg [22].

#### 5.1.6. Doramectin, Eprinomectin, and Nemadectin

Doramectin has been used as an injectable (10 mg/mL) or topical form (5 mg/mL) in ruminants, pigs, and cattle [34,35]. Eprinomectin is poured on cattle at a concentration of 5 mg/mL and for treating *Toxocara canis* in dogs at a dose of 0.1 mg/kg [36]. Nemadectin is used to treat gastrointestinal helminths in dogs at doses of 0.2 to 0.6 mg/kg [37]. Animals with *ABCB1* gene defect have been shown to develop hypersalivation, bradycardia, slow mobility, restlessness, blindness, and depression. In normal animals, similar clinical signs for epinomectrin and nemadectin overdose are expected. However, the particular dose to be termed overdose has not been determined [38]. The chemical structures, synonyms, IUPAC name, and molecular formula of ivermectin, selamectin, moxidectin, moxidectin, eprinomectin, nemadectin, abamectin, milbemycin and doramectin are shown in Table 1.

### 5.2. Dosing

A commonly used therapy in recent times has been based on oral, parenteral, topical, or spot topical (as in veterinary flea repellant “drops”) administration of avermectin. They show activity against a broad range of nematodes and arthropod parasites of domestic animals at doses of 300 μg/kg or less than 200 μg/kg of ivermectin appears to be the common interspecies standard, from humans to horses to house pets, unless otherwise indicated. Unlike the macrolide or polyene antibiotics, they lack significant antibacterial or antifungal activity [40]. Taylor et al. [41] illustrated an annual single oral dose of 150 μg/kg of ivermectin was given to prevent microfilarial production and inhibit disease progression. In humans, the most commonly used dose of ivermectin varies from 150 to 200 µg/kg for the treatment of enterobiasis onchocerciasis, and strongyloidiasis, while a higher dose of 400 µg/kg was used for lymphatic filariasis. It is worth noting that ivermectin was administered twice a week, up to 1.6 mg/kg subcutaneously, for patients with spinal damage and muscle spasms, for 12 weeks [42]. Avermectin IB1 demonstrated the potential to minimize tumor growth at a dose of 1 mg/kg in SHK male mice with a strong Ehrlich carcinoma as well as human acute myeloblastic leukemia, breast and colon carcinoma, glioblastoma, and murine lymphosarcoma cell line. The median dose used was 5 mg/kg (2.4–40 mg/kg), equivalent to 0.40 mg/kg in humans [43,44,45,46]. A dose of 150 μg/kg of ivermectin was reported to be linked to reduce mortality rates and lower healthcare resource use in COVID-19 patients. [47,48]. Laing et al. documented that a single dose of 30 µg/kg of ivermectin significantly reduced the number of skin microfilariae, and that effect continued for at least 6 months without any significant adverse effects [49].

#### 5.2.1. Mechanism of Action

**Figure 2:**
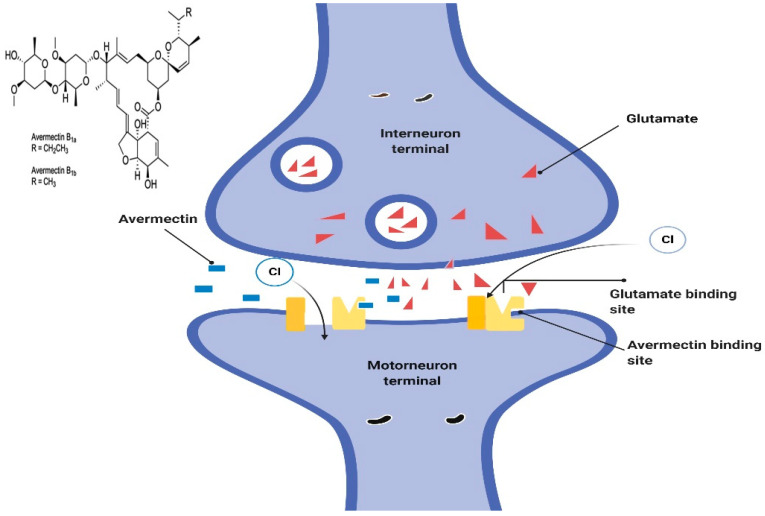
The proposed model to describe mechanisms of ivermectin sensitivity.

Avermectin generally works by preventing the transmission of electrical impulses in the muscles and nerves of invertebrates by amplifying the glutamate effects on the invertebrates-specific gated chloride channel [50,51]. This allows more chloride ions to enter the cells, causing hyperpolarization and culminating in paralysis of the invertebrate neuromuscular systems. The administered doses that lead to this damage are not toxic to mammals, the reason being that they lack the glutamate-gated chloride channel [52] (Figure 2).

Low concentration of ivermectin does not destroy the worms by its effect on GluCls expressed in the neurons, as hyperpolarization caused by ivermectin spreads through gap junctions encoded by unc-7 and unc-9 to other excitable cells important for the worm’s activity. A study in GluClα3 (AVR-14) and GluClα1 (GLC-1) to confer sensitive gap junction to ivermectin has shown that it is important for hyperpolarization to spread from the extra pharyngeal nervous system to the pharynx [53]. This process occurs by connecting neurons, such as I1 and RIP, which may not be expressed by GluCls [28,54]. The information on the role of avermectins on chloride channels opening, decreasing muscle fiber’s resistance to input, GABA-like effects, and nerve signal transmission has been well documented [55]. Some early results on different species, various model systems, and at varying concentrations are as follows: stimulation of high-affinity binding of GABA and benzodiazepine to rat brain membranes. Listed below are some of the experiments that were conducted using various types of species, different experimental designs, with different doses. A quick disabling of nematodes devoid of hypercontraction or soft disability. A very low concentration of avermectin induced a quick and fast irreversible inhibition of inhibitory postsynaptic potential in the nerve of crustacean and delay reducing the excitatory potential amplitude and elevating the chloride ion entry. High avermectin induced by membrane conduction was inhibited using GABA antagonists (e.g., bicuculline and picrotoxin) [56]. The signal transmission between ventral interneurons and motor neurons of Ascaris was blocked. The nanomolar concentration of avermectin in the extensor tibiae muscle of the locust *Schistocerca gregaria* has been reversibly increased by the chloride ion in the GABA sensitive fibers. Micromolar concentrations of avermectin also reversibly prevent muscle fibers from *Schistocerca gregaria* that are either sensitive or insensitive to GABA [57]. Another study showed that subpicomolar concentration of avermectin induced reversible opening of multi-transmitter-gated chloride channels isolated from the stomach muscle of crayfish using a patch-clamp technique. It should be noted that the chloride channel can be activated using GABA, glutamate, or acetylcholine [58]. This experimental design was able to distinguish between two different conductance states, the first being activated by carbachol, glutamate, ibotenic and quisqualic acid, while the second was activated by GABA and muscimol (a GABA agonist). Avermectin was not effective in activating the second conductance like GABA. This is because the effect is direct and thus it does not require a second messenger pathway. However, a huge non-reversible increase in the opening of chloride channels occurs when the avermectin concentration increased to or above 10 pmol and this reversible or nonreversible opening of the chloride channels was blocked by picrotoxin [58]. Further studies were carried out on the effect of ivermectin on the electrophysiology of the oocytes obtained from Xenopus laevis. A 1–2.5 kb size of poly (A+) RNA obtained from *C. elegans* was injected into the oocytes. The results showed that the induction of inner-bound membrane stream associated with high ivermectin concentration as well as increasing the entry of chloride ion across the membrane [59]. The result also shows that GABA, muscimol, benzodiazepam or bicuculline do not alter the effect of ivermectin, while picrotoxin does. Based on the above-mentioned results, it can be concluded that ivermectin can directly induce the GABA-insensitive chloride channels opening [59,60]. To understand the mechanism by which avermectin acts against different species of insects, arthropods, nematodes and immature worms such as flukes, tapeworms and filarial worms, couples with good tolerance by the host animals. It is necessary to isolate the binding site of avermectin in various species and study how it induces the opening of chloride channels and increases hyperpolarization, since it has been established that its binding site is specific to other effector molecules [61].

#### 5.2.2. Mode of Action of Milbemycins

The mode of action of milbemycins is not well understood compared to avermectin although its anti-parasitic properties have been discovered and described more than two decades ago [62]. Nemadectin is the most potent milbemycin that has unsaturated long-chain C at its 25-position. Nemadectin has been shown to possess insecticidal and nematocidal activities. It also serves as a substrate for moxidectin (MOX) synthesis, a commercial endectocide [63]. As with other macrocyclic lactones, there is a high affinity for invertebrates-specific glutamate-gated ion channels. The glutamate-gated binding site exists in juxtaposed to GABA-gated chloride channels, therefore, there is a possibility of macrolide endectocides enhancing GABA-gated chloride channels. MOX also binds to the myroneural junctions of anthropoids and neuronal membrane of nematodes [64]. Hyperpolarization of the postsynaptic cells occurs as a result of the influx of chloride ion, which decreases the resistance of the cell membrane. This disrupts the neurotransmission processes, causing disability, death, and elimination of the parasite [65]. It has also been reported that MOX inhibits the release of dopamine from the striatum due to the activation of the GABAergic system, leading to reduced motor coordination in rats [66].

#### 5.2.3. Mode of Action of Spinosyns

Unlike other avermectin compounds, spinosyns elicit its insecticidal activity through a different mechanism [67]. Several reports suggested that spinosyns may alter neuronal function by affecting the function of the nicotinic receptor [68,69]. The nicotinic-receptor binding site for spinosyn A in insects, spinosad, has not been identified, including the GABA or nicotinic binding site for insecticide. In addition, spinosyn does not interact with the binding site for avermectins [70]. A study carried out by Robertson et al. [71] showed that spinosyn A induced nicotinic effect, suggesting an unknown mechanism by which it binds to the nicotinic receptor. A new study using a knockout *Drosophila melanogaster* suggested that Dα6 subunit of the nicotinic receptor as a potential binding site for spinosyn [72].

## 6. Pharmacological Effects of Ivermectin

### 6.1. Human Uses of Ivermectin

The origin of ivermectin use in the treatment of human diseases started in 1985, in an effort by the united nation to tackle the challenges posed by tropical diseases, which have become endemic in various areas of Africa. Some of these diseases that are treated with ivermectin, including onchocerciasis, caused by a filarial nematode, *Onchocerca volvulus* [73]. Presently ivermectin is used to treat strongyloidiasis, scabies, pediculosis, gnathostomiasis, myiasis, and leishmaniasis [74,75]. In addition, other studies have shown that ivermectin can be used to reduce the prevalence and spread of other infectious diseases linked to a helminthic parasites transmitted through soil such as strongyloidiasis, ascariasis, trichuriasis, and enterobiasis hookworm, Trichuris and Ascaris. These diseases have been identified as major cause of death for underdeveloped children with poor nutrition and retarded growth [76,77]. Topical administration of ivermectin to children also reduced the prevalence of head lice and scabies [78]. Moreover, ivermectin has also been shown to be effective in the treatment of *Papulopustular rosacea* (PPR), a human skin disease characterized by transient pustules and facial erythema [79]. In order to enhance the efficacy of ivermectin in the treatment of infectious diseases in human, it has been administered with other drugs. For example, ivermectin is combined with doxycycline to inhibit onchocerciasis transmission in humans [80]. Other study showed that ivermectin combined with doxycycline has also been shown to improve the efficacy of ivermectin in suppressing microfiladerma, as it has been shown to reduce the symbiotic endobacteria of filariae, Wolbachia spp. that is important for the reproduction and survival of mature female worms [81]. Moreover, another study stated that the daily administration of ivermectin-antibiotic combination for six weeks was more effective in reducing the microfiladerma levels in infected patients than ivermectin alone. The antiparasitic activity of ivermectin in the treatment of onchocerciasis was also improved when combined with amorcazine compared to ivermectin itself [82]. The study also showed that ivermectin has the potential to be used in the treatment of mites and human lice infestation [83]. Ivermectin has also been reported to possess other therapeutic properties such as filaricidal in the lymphatic system and as an anticonvulsant against lidocaine- and strychnine-induced convulsion [84]. The anticonvulsant properties of ivermectin is linked to the activation of a receptor-ion channel that is independent in the glycine and strychnine pathway [85]. Moreover, Kane et al. [86] revealed that the anticonvulsant effect of ivermectin is mediated by GABAA receptors. The summary of human uses of ivermectin is shown in Table 2.

### 6.2. Efficacy against Plant Parasitic Nematodes

One of the commercially available forms of avermectin is abamectin, which contains 80% avermectin B1a and 20% avermectin B1b. It is reportedly to be used either as an insecticide or acaricide in several countries, mostly in countries that depend on agriculture and animal husbandry for preservation and protection, respectively [87,88]. In addition, Khalil et al. [32] reported its use as a protective agent against plant nematodes that feed on the roots, during seed germination, coupled with its long shelf-life, while Hussein and Sabry [89] reported its molluscicides activity against matured *E. vermiculata* in the growth of wheat. Another avermectin product, emamectin benzoate, a semi-synthetic product of avermectin has been shown to be very active as a nematocide in destroying root-knot nematodes when administered at the nursery bed of chili. It significantly reduced the population of nematodes meloidogyne spp.) in a dose-dependent manner. Avermectin has been reported to be effective against other nematodes including: *Hoplolaimus galeatus* and *Tylenchorynchus dubius* [90]. Several reports have confirmed the efficacy of avermectin as a nematocides against various nematodes that attack plants. Jayakumar et al. [90] reported the effectiveness of avermectin in the treatment of seed against reniform nematode, and *Rotylenchulus reniformis* used crude avermectin to treat seed. Blackburn et al. [91] also reported the nematocide effect of avermectin B1 in reducing the population of *H. galeatus* and *T. dubius*. In an experiment conducted by Das et al. [92], the efficacy of emamectin benzoate as a nematocide in the reduction of root-knot nematode (Meloidogyne spp.) populations was reported when used in the nursery bed of chili.

### 6.3. Antibacterial Action

An observed non-prescriptive activity of avermectins is its antibacterial activity. The primary pharmacological activity of avermectins is its use as insecticides, nematocides, antihelminthic and arachnicides [93]. However, recent studies have shown that avermectin is not effective against broad spectrum bacteria, others have shown it is effective against some bacteria species [93]. Some avermectins have been evaluated for antibacterial activities include ivermectin, selamectin, doramectin, and moxidectin. Using the approved standard methods based on the gold standard proportion, glycerol reuptake or nitrate reductase, resazurin of viable colony forming unit after exposure [94]. The 3 (4, 5-dimethylthiazol-2-yl) 2, 5 diphenyl tetrazolium bromide (MTT) assay method was used to test the antibacterial activity of four different avermectins in different *Mycobacterium* species. All four were effective against both *Mycobacterium bovis* and *M. tuberculosis* laboratory strains (H37Rv, CDC 1551, and Erdman) in a concentration-dependent manner. While all of them were effective against *M. smegmatis* apart from doramectin. They showed a lower inhibitory effect against *M. avium* [95]. With a recent study showing that the presence of glycerol in the assay medium plays an important role in the antibacterial activity of some drugs on some mycobacterium species, the inhibitory activities of selamectin, moxidectin, and ivermectin were only slightly affected in the absence of glycerol in the assay medium [93,96,97]. Lim et al. [93] also tested the antibacterial activity of avermectins on some *M. tuberculosis* that are resistant to drugs (multidrug-resistance [MDR] and extensive drug-resistance [XDR]. Twenty-seven MDR and XDR isolated from different geographical location have been confirmed to be resistant to anti-TB drugs such as kanamycin, ethambol, rifampin, ethionamide, rifabutin, isoniazid, p-aminosalicyclate (PAS), streptomycin and pyrazinamide. All were sensitive to MIC activity of avermectins except three MDR and two drug susceptibility. Clinical trials of selamectin, moxidectin, and ivermectin have shown that their antibacterial activity is bactericidal against *M. tuberculosis* strain H37Rv and mc^2^ 587. They all reduce the number of the initial bacteria viability up to six orders of magnitude [93]. Selamectin is the most potent with respect to bactericidal activity, while ivermectin was the most potent bactericidal avermectin against MDR strain. The sensitivity of *Mycobacterium* spp. to avermectin as compared to other bacteria might be linked to the nature and complexity of their cell wall envelope [98]. As stated earlier by Wolstenholme and Rogers [99], avermectin causes paralysis and fecundity by binding to glutamate-gated chloride channels on the muscles and nerves of the parasite, which does not occur in humans due to the lack of different channels that bind to avermectins. Observations that selamectin and moxidectin, majorly used as an antiparasitic drugs in animals with a report of no toxic effect, showed a strong activity against *M. bacterium*. This shows that avermectins, with their high specificity for some mycobacterium can be selected for some pathogens that are resistance to antibacterial drugs, without any concern for side effect on the intestinal gut flora when administered orally. The study also showed that ivermectin and moxidectin can also inhibit the growth of different strains of *M. ulcerans* at concentrations ranging from 4 to 8 μg/mL. While they are not effective in *M. marinum* which shares similar features with *M. ulcerans.* They also found out that IVM performed better than the standard drug rifampicin at the administered MIC concentration. And when the two drugs were combined (IVM and rifampicin), a better activity was observed as compared to individual activity in eliminating the parasite [99]. It was suggested that the induction of P-glycoprotein/ABCB1 in human by rifampicin may reduce the plasma concentration of IVM. Of all the types of avermectin tested against *M. ulcerus*, that causes Buruli ulcer (BU) in vitro, selamectin and milbemycin were the most potent with MIC concentrations of 2 to 4 μg/mL and 2 to 8 μg/mL, respectively, while IVM and moxidectin were not significantly active (MIC > 32 μg/mL) overall, it was concluded that selamectin had the best potential in the treatment of BU. Besides, of all the tested isolates of *Staphylococcus aureus* (>20) using IVM, only 2 isolates were sensitive to IVM [94]. The LD_50_ of IVM in animals is 50 mg/kg, while the administered dose in animals and humans is 200 μg/kg and peaked at the concentration of 52 ng/mL in plasma. The potential of IVM in the treatment of methicillin-resistance and methicillin-sensitive *S. aureus* isolates can make IVM a good antibiotic in addition to its anti-helminthic properties [94].

### 6.4. Endectocides for Malaria Control

Malaria is one of the top diseases that cause death among infants, especially in India and Africa. It is caused by *Plasmodium falciparum* in Africa and transmitted to humans by female anopheles. Progress has been made in reducing malaria deaths since 2010 [100]. However, recent data showed that there is substantial rise in the number of malaria deaths. It was noticed that the major challenge in the management of malaria is the complexity in the life cycle of the parasite, which often develops resistance to various approved drugs [101]. The insecticidal activity of ivermectin and its specific effect on *Anopheles* mosquito prompted an investigation into its antiplasmodial activity against the malaria parasite in order to use it for malaria treatment [102]. The antiplasmodial and insecticidal effects of ivermectin often depend on the concentration, route of administration, duration, species type, and experimental design [103]. Ivermectin was reported to inhibit the development of *P. falciparum* at the asexual stage with IC_50_ in the range of 1–10 µg/mL, however, ivermectin had no effect at concentrations of 3.9–1000 ng/mL on *P. vivax* [104]. However, incubation of ivermectin with a different dilution of *P. vivax* with plasma from healthy individuals (4.28–34.24 ng/mL) for 4 h, ivermectin was able to inhibit the growth of *P. vivax*. It is reported that the metabolite of ivermectin may be responsible for the inhibitory activity of ivermectin at low concentration. The lack of ivermectin effect on *P. falciparum* in vitro might be due to the absence of the glutamate-gated chloride channel and GABA-gated chloride channels in *P. falciparum* where ivermectin binds to insects [104,105] it was also reported that ivermectin has no significant effect on *P. falciparum* at the blood stage of development in vitro, while the possible mechanism of ivermectin in inhibiting the parasite at the blood stage might be by blocking nucleo-cytoplasmic shuttling of *P. falciparum* signal transduction particle (SRP) components [104]. This process takes place through a cascade of events involving the induction of chloride dependent polarization of the *Plasmodium* nuclear membrane then altering the shuttling of importin α/β, which leads to the inhibition of SRP polypeptides [106]. It is believed that the importin α/β might be karyophyrin α/β as it is the only importin present in *P. Falciparum*. A different observation on the process by which ivermectin inhibits the growth of mosquitoes when infected with *P. falciparum* [106]. Kobylinski et al. [103] reported that ivermectin decreased the intensity and prevalence of oocyst in different mosquitoes, while de Carvalho et al. and Perez-Garcia et al. [104,107] reported a reduction in the prevalence of oocyst without affecting the intensity in mosquito infected with *P. falciparum* and *P. vivax*. Three different avermectins were also tested with respect to the hepatic effect of *Plasmodium*. Ivermectin, eprinomectin, emamectin were orally administered into mice at 10 mg/kg thrice, using soybean oil as a vehicle before infection with *P. berghei* 24 h later. The parasite load was determined between 44–46 h post infections. It has been found that ivermectin has been shown to be effective with approximately 88% protection against liver infection while the other two have been shown to be ineffective compared to primaquine, the standard drug [106]. Of note is the mild neurotoxicity of ivermectin in some of the mice treated. One of the major observation in a mouse-model of antimalarial activity of ivermectin showed mild neurotoxicity as impairment in infection and behavior were observed in the mice. With the potent insecticidal activity of ivermectin against female Anopheles mosquitoes, it was concluded that the ability of ivermectin to prevent parasite invasion of the liver could be central to malaria treatment and prevention as it decreases the level of *Plasmodium* in the blood and other tissues [108]. A study by Mekuriaw et al. [109] showed that ivermectin has anti-plasmodial activity in humans infected with An. Arabiensis which might be linked to the delay effect on fecundity. More experiments still need to be conducted on the efficacy of ivermectin in treating malaria.

### 6.5. Anti-Inflammatory Effect of Avermectin

New discovery on the pharmacological activity of ivermectin is its potential usage as an anti-inflammatory drug. Ivermectin has been reported to modulate immune activity in mast cells and macrophages that are important in T-cell mediated skin inflammation through the following process: (1) priming of T cell through skin emigration of allergen-loaded DC [110]. (2) Recruitment of effector T cells by the skin, and (3) generating a cluster of T-cell/DC through macrophage, this is important in dermal skin inflammation [111]. Ventre et al. [112], using murine, showed that application of ivermectin topically delays skin inflammation as a result of allergy prompted by regular contact with Dermatophagoides farina (an allergen), slowing the synthesis of inflammatory cytokines, priming and activation of allergen-specific T-cells. They also showed that ivermectin did not have a significant effect on dendritic cells functions, either in an in vivo or in vitro, but caused impairment in the activation of T-cells, production and proliferation of cytokines by stimulating antigen-specificity and polyclonal activity. Nörenberg et al. [113] and Wareham et al. [114] showed that ivermectin possess immunomodulatory activity in mast cells and macrophages in various in vitro models. Thus, the anti-inflammatory effect of ivermectin can be linked to its regulatory effect in T- cells, skin mast cells and macrophages. Leyva-Castillo et al. [115] also showed that ivermectin -induced modulation of T-cells does not involve some target expressed by T cells such as P2XR4 (involved in the regulation of Ca^2+^metabolism) and FXR (a receptor for which ivermectin has a strong affinity for). However, other receptors involved in T-cell proliferation and screened in vitro showed that they were also not involved in IVM-immunomodulatory effect on T-cell. Some of these receptors include Ryanodine receptors (RyRs), gamma aminobutyric acid (GABA_A_Rs) type A receptors, type 3, 5-hydroxytryptamine receptors (5-HT3Rs) [116]. In addition, Ci et al. [117] showed that the mechanism of anti-inflammatory activity of avermectin might involve a reduction in the pathway of mitogen-activated protein kinase (MAPK) and nuclear transcription factor kappa B (NFkB).

### 6.6. Anticancer

Another non-prescription discovery for ivermectin is as an anticancer drugs. The ability of ivermectin to stimulate the intracellular influx of chloride ion in cells from a leukemia patient and induction of oxidative stress leading to the death of the leukemia cells without having any effect on normal cells [43]. The anticancer effect of ivermectin against leukemia was also tested in mice using three different models, all of which demonstrated the ability of ivermectin to inhibit or slow the growth of tumor cells. Most tissue cancers in human have a deregulated WNT-TCF (WNT-T cell factors) signaling pathway. This pathway regulates cell proliferation and metastasis. The antitumor effect of ivermectin in human colon cancer and lung carcinoma xenograft is dependent on WNT-TCF signaling pathway, it is effective if the cancer is WNT-TCF-dependent and ineffective if it is not [118]. The potency of ivermectin as an anticancer in a glioma xenograft is also observed by inhibiting DEAD-vox RNA helicase DDX23 [119]. This type of helicase is linked to miR-21 processing, a microRNA associated with glioma the proliferation and metastasis of glioma cells. One of the key pathways in colon cancer is the inhibition of TGFβ-R2 (a WNT-TCF repressor) by miR-21, leading to the overexpression of WNT-TCF [120]. This shows that ivermectin can play a dual role in preventing tumor growth and metastasis. Ivermectin can also inhibit the depolymerization of microtubules, thus preventing mitosis in tumor cells as well as increasing the polymerization of microtubules [121].

### 6.7. Metabolic Effect of IVM

Ivermectin has also been reported to affect lipid and carbohydrate metabolism in mammals by targeting farnesoid X receptor (FXR), a nuclear hormone receptor that is involved in glucose, cholesterol, and bile control. Ivermectin binds to FXR to initiate its transcriptional function and the recruitment of other metabolic regulators. An experimental model of diabetes using mice shows that ivermectin significantly reduced serum glucose and cholesterol concentrations and enhanced the sensitivity of insulin, however, ivermectin had no effect in FXR-null mice [122]. *C. elegans*, a nematode in which ivermectin is active against, possesses a DAF-12 (a nuclear hormone receptor that regulates its life cycle), that shares almost similar homologue with FXR [123]. However, there is no proof if ivermectin elicits its nematocidal activity through this receptor.

### 6.8. Anti-Alcohol Therapies

Four different types of AVM were tested for their potential as anti-alcohol therapy, namely selamectin (SEL), abamectin (ABM), ivermectin (IVM) and moxidectin (MOX). IVM modulates the action of ethanol on P2X4Rs in an in vivo study, which may be associated with a decrease in alcohol intake [124]. Also in both male and female mice of the C57BL/6J strain. IVM at a dose between 1.25–10 mg/kg reduces alcohol intake in various models mimicking different types of alcohol intake in human such as binge drinking, social drinking, and alcohol-induced behaviour [125,126]. IVM elicits its anti-alcohol effectively at 9 h post-administration, a time that us close to the half-life of IVM in the plasma [127]. It also showed that the least effective dose of IVM (2.5 mg/kg) is in proximity to the level of IVM in the brain, which is 0.28 ng/h/mg tissue. This shows the decrease in alcohol intake can be linked to the administration of IVM. IVM does not have any significant effect on water and food intake as well as weight change and general physiology of the mice. Using a self-operant chamber technique designed by Yardley et al. [127], IVM also caused a reduction in preference for alcohol bottle and appetite for alcohol consumption. This same observation was reflected in another experiment using both male and female rats of HAD-1 and HAD-2 strains [128]. Proving that IVM can be effective as an anti-alcohol therapy in different animal models of alcohol intake. Another model representing the long-term exposure of IVM to alcohol intake was conducted by [127,129]. IVM was administered intraperitoneally to female C57BL/6J mice strain at a dose of 2.5 mg/kg for seven consecutive days and resulted in a substantial decrease in alcohol intake as well as the option to drink alcohol bottle or another bottle, the mice preference reduced with respect to alcohol bottle [127]. Yardley et al. [129] using the same female mice strain as Yardley et al. [127], also administered IVM at 3 mg/kg for 10 consecutive days and also reported a reduction in alcohol intake without affecting fluid intake or body weight. The various models used a dose extrapolated from the approved pharmacology dose for humans. Another experiment to see whether modifying the route of administration of IVM would still produce the same anti-alcohol effect of IVM was conducted by [126]. The oral administration of IVM for 30 days also shows similar findings with i.p administration. They also show that IVM did not cause any histological changes in the kidney, liver, and brain tissues of the animals. Neurobehavioral effect of IVM has also shown that the drug does not have any neurological deficits, such as perceptual, cognitive, and emotional functions, however, mild anxiolytic activity was observed, which is likely linked to the GABA_A_Rs potentiation [130]. A clinical study involving administration of 120 mg of IVM to humans did not induce any adverse reaction or toxicity [131]. In summary, IVM has shown a great potential to be used as an anti-alcohol drug with minimal adverse effects. To study the mechanism by which IVM can act as an anti-alcohol drug, Natsuaki et al. [111] conducted various in vitro and in vivo experiments. It has been discovered the rate of alcohol consumption significantly reduced in male P2X4R knock-out mice, hypothesizing that the anti-alcohol potential of IVM may be related to the potentiation of P2X4R. Jin et al. [132] showed that ethanol addiction can be linked to its ability to inhibit pre-synaptic P2XRs in the synaptic terminals that releases GABA in the VTA, preventing the release of GABA into the DA neurons, which inadvertently lead to an increased firing of VTA and DA neurons, suggesting that the addictive effect of ethanol can be countered by DA system inhibition. Khoja et al. [133] also provided more evidence of a link between DA system and IVM. Their findings showed that IVM can play a role in the phosphorylation of cAMP response element binding protein (CREB) and DARP-32 (a 32 KDa phosphoprotein) as well as dopamine in the ventral striatum though P2X4R potentiation. Based on a previous study by [134,135] that reported a link between coordination of ethanol-linked behavior and phosphorylation of CREB and DARPP-32, Khoja et al. [133] concluded that the mechanism by which IVM acts as an anti-alcohol effect of IVM might be by controlling steps linked to dopamine signaling in the brain area involved in drug reward circuitry. Abamectin, another type of AVM, shares similar structure with IVM, the difference being the presence of a double bond between the carbon atom at positions 22 and 23 of the spiroketal unit. Asatryan et al. [125] showed that ABM had similar effect on Xenopus oocytes as observed with IVM. They also find out that ABM enhanced P2X4R potentiation more than IVM at higher concentrations and acting as a direct agonist in P2X4R potentiation in comparison with IVM, as it enhances P2X4R activity in the absence of ATP. There was no difference in the potentiation of GABAARs-mediated current between ABM and IVM, however, the potency reduced with increased concentration when compared to IVM. Their results also showed that ABM reverses the inhibition of ATP-activated current by ethanol, decreased ethanol intake and enhanced water intake. The variance between the anti-alcohol effects of ABM and IVM was not well explained, suggesting more research on their mechanism. SEL, another type of AVM showed low ow P2X4R potency and reversed ethanol inhibitory activity compared to IVM and ABM. Lespine et al. [136] reported that the poor activity of SEL can be linked to its structural variation. SEL contains only one saccharide group, which is believed to limit its affinity for p-glycoprotein [136]. Apart from this, other differences between SEL and IVM includes, replacing the isobutyl and alicyclic hydroxyl groups in IVM with a cyclohexyl ring and an unsaturated ketoxime group in SEL respectively. Asatryan et al. [125] reported that very high amount of SEL (10 µM) was required to potentiate P2X4R as compared to IVM (0.5 µM), a weaker action on GABAARs as compared to IVM and ABM, leading to its inability to antagonized the inhibitory effect of ethanol on P2X4Rs. They also reported the inability of SEL to affect appetite or preference for ethanol intake. MOX is a type of AVM that is structurally different from IVM [137]. The isopropyl group in IVM is substituted by dimethyl butyl group and MOX contains a methoxime group from which its name was derived. These features are believed to enhance its lipophilicity and might increase its ability to cross the blood brain barrier (BBB). Another difference between MOX and IVM is that P-gp has low affinity for MOX, which means that MOX does not allow P-gp to be extracted from the brain. In addition, it also does not have a significant effect on GABAARs [138,139]. The advantage of these differences is that it reduces the potential toxicity as a result of accumulation from long-term usage in brain deficient in P-gp and effect that IVM has on the brain and interaction of MOX with other drugs that stimulate GABAARs which might lead to depression and coma. Result also showed that MOX at varying concentration induces currents in Xenopus oocytes via the potentiation of P2X4Rs significantly and reverse the inhibitory effect of ethanol at low concentration but had no effect at high concentrations [139]. In vivo study also showed that MOX reduced the desire for ethanol consumption in C57BL/6J mice of both sexes in different models of alcohol intake [139]. Comparing the various doses used for IVM, it has been shown that MOX was more efficient on reducing ethanol consumption as it takes 4 h for MOX to reduce ethanol intake, while IVM takes a longer period to exert similar effect.

### 6.9. Antitumor Effect of Avermectins

Drinyaev et al. [140] were the first to confirm the antitumor activity of AVM. They tested the combination effect of AVM C and AVM B on tumor growth using experimental mice. It inhibits the growth of 755, ascites Ehrlich and solid Ehrlich carcinoma and P388 lympholeukemia, and the highest inhibition of tumor growth (70–80%) observed when administered intraperitoneally. AVM have also been shown to improve some anticancer drugs (e.g., vincristine) as they synergistically increase the suppression of the growth of EC, P388 lympholeukemia, and melanoma B16 when administered after vincristine injection. IVM has been identified as one of the avermectin group of drugs that has the potential to treat leukemia cells [43]. IVM was reported to be effective in cell death induction of myeloid leukemia at low concentration without affecting normal hematopoietic cells. In three independent experiments involving the induction of leukemia in mice, IVM slows the growth of tumor cells at a dosage that seems replicable in humans. This antitumor activity is believed to be linked to the established ability of IVM to increase chloride influx in parasite and nematodes, as IVM increased the concentration of chloride ion and cell polarization in leukemic cells. In addition, IVM increases reactive oxygen species (ROS) in leukemic cells, which may contribute to the cytotoxicity observed in leukemic cells [141]. It was also reported that IVM enhances the antitumor effect of established drugs such as cytarabine and daunorubicin. A low concentration of IVM has been reported to alter the tumor growth through a constitutive mechanism involving direct activation of transcriptional activity of TCF, repressing the phosphorylation of β-catenin and cyclin D at the C-terminal [142]. IVM was also revealed to be active as an antitumor agent in human colon cancer xenograft and lung cancer in an in vivo experiment through inhibition of TCF and blocking of WNT-TCF pathway without any side effects [142]. There are reports that the mode of action of avermectin in tumor cells might be different from that of nematodes and parasites. As stated earlier, the antitumor activity of AVM drugs may be due to the transcription pathway of TCF, phosphorylation of cyclin D and β catethin, whereas, in the parasite, it is through the chloride-gated ion channels. Some AVM, such as moxidectin, have been reported to be ten times more active than IVM as nematocides, whereas this same moxidectin is less active or not active at all in human cancer cells. A particular concentration to cause behavior is observed for each measurable effect of IVM. Cytotoxicity of tumor cells is achieved at a concentration above 10 μM or longer while for 48 h or longer if the concentration is above 5 μM. For the inhibition of TCF transcription and cell death induction through apoptosis, the concentration is lower at different durations [43]. With respect to a brain tumor, the use of IVM may require caution as the blood brain barrier features might have been lost, leading to the entry of IVM into the brain. The brain contains the gated-chloride ion and GABA-channels that IVM binds to in nematodes, thus IVM might bind to them in the brain leading to brain injury and death in normal cells [143]. Melotti et al. [144] summarizes the mechanism of antitumor activity of AVM, such as ivermectin, selamectin, and other types as follows: detects the role of avermectin B1 in inhibiting the activation of WNT-TCF reporter activity by N-terminal mutant (APC-insensitive) β-CATENIN as detected in their screen; detects the ability of AVM B1, IVM, doramectin, MOX and SEL to parallel the modulation of WNT-TCF targets by dnTCF; the finding that the specific WNT-TCF response blockade by low doses of Ivermectin and Selamectin is reversed by constitutively active TCF; the repression of key C-terminal phospho-isoforms of β-CATENIN resulting in the repression of the TCF target and positive cell cycle regulator CYCLIN D1 by IVM and SEL; the specific inhibition of in vivo-TCF-dependent, but not in vivo-TCF-independent cancer cells by IVM in xenograft. The study showed that the regulation of phosphorylation of β catenin may be by a ser-552 or ser-675 on the catenin polypeptide chain. The PP2A/PP1 protein phosphatase-blocked situation may be used by IVM or selamectin to improve this step either directly or indirectly to dephosphorylate P-ser552 / P-ser675. This can be further explained in the relationship between β-catenin and TCF factors as an important step in WNT signaling in human colon cancer cells [145]. The decrease in the concentration of phosphorylated β-catenin in colon cancer cells also requires the Bα (PR55α) subunit of PP2A [146]. This shows the complex nature involved in the inhibition of β-catethin phosphorylation and a potential multiple targets of IVM as an anticancer agent in colon cancer [147]. One of the important information from these findings is that the human colon cancer cell lines are TCF-dependent [148]. For cancer cells deficient in the APC destruction complex, it is predicted that the pathway involved in the IVM-induced enhancement of PP2A is silent. IVM treatment is also reported to reduce some important markers of colon cancer cells, such as *ASCL2* and*LGR5* [149,150]. The dependent of cells on TCF also on the environment of the cell. For example, it was reported by [148] that Ls174T and primary CC1_4_ cells are TCF-dependent in vitro, while in an in vivo condition, they turn to TCF-independent cells. Although the mechanism involved is not yet understood, de Sousa et al. [151] reported the role of DNA methylation in the process involved in the switch over. However, some colon cancer cells, such as DLD1 remain TCF-dependent either in vitro or in vivo [148]. This is one of the proofs of IVM safety as an anticancer drug, as it only targets TCF-sensitive cancerous cells in vivo and not normal cells at the administered dose. However, non-specific toxicity might be developed at higher dosages. Apart from anticancer usage of IVM, it can also be used as a prophylactic drug against some TCF-dependent intestinal tumor and sporadic colon tumor in an aging population.

### 6.10. Antiviral Effects

The role of IVM in inhibiting RNA helicase has led to research on the potential of AVM in the treatment of viral-related diseases. IVM has been reported to inhibit the replication of most flavivirus through blocking viral helicase [152]. Some of the diseases caused by flavivirus include dengue, yellow fever, tick-borne encephalitis, and West Nile virus. This led to the submission of a patent application for IVM as an off-label for antiviral therapy in humans. A study also showed that consistent passaging of IVM for a six months duration did not induce resistance in the yellow fever virus, suggesting that the helicase domain may not be able to undergo mutation [28]. In addition, the report revealed that IVM had no activity against other types of virus, though it inhibits the replication of HIV-1 and dengue virus at high concentrations of IVM (25–50 μM) in an in vitro experiment. Nuclear import of viral proteins is critical to the life cycle of many viruses, including many RNA viruses that replicate exclusively in the cytoplasm such as DENV, respiratory syncytial virus, and rabies [153,154,155,156]. The mechanism of inhibition of viral replication is linked to its inhibition of importin α/β-mediated transport, leading to the alteration of the trafficking of viral protein between the host cell cytoplasm and the nucleus [157,158]. Forwood and Jans. [159] also discovered that IVM prevents the ability of SV40T-ag to recognize Impα/β1.,this observation led investigators to subject IVM to various nuclear-localizing proteins such as IN, T-ag, hCMV UL44, p54, Impα/β-recognized protein, and Impβ1-recognized proteins in HeLa cells and compared with a standard drug (mifepristone). The result showed that IVM inhibited the accumulation of IN nuclear and T-ag nuclear, while mifepristone inhibited the accumulation of IN nuclear only [160]. They also showed that IVM only inhibits nuclear protein that contains Impα/β1-recognized, as it did not inhibit TRF1 SRY or PTHrP which are transported in the nucleus dependent of Impα/β1 [161,162]. From their results, it can be suggested that IVM is specific for a particular type of Impβ-recognized nuclear import, called Impα/β1-recognized nuclear import cargoes, and has no effect on other nuclear import pathways. In addition to the above report, IVM does not affect histone H2B and SUMO-conjugating enzyme UBC9 which are both imported into the nucleus by multiple different Impβ homologues and Imp13 respectively [163,164]. The antiviral activity of IVM and ribavirin was also tested against Newcastle disease virus (NDV) using a 9-day old chicken [165]. They observed that IVM and ribavirin were cytotoxic at concentrations above 50 μg/mL and 12.5 μg/mL, respectively. However, the most potent concentration of IVM to inhibit virus growth is from 100 μg/mL and above, while the antiviral activity of ribavirin was experienced at all concentrations (6.25–200 μg/mL). The antiviral activity of IVM against HIV is also linked to an importin (IN) protein. HIV produces a complex called PIC (pre-integration complex), made up of a newly transcribed viral cDNA, IN, host proteins, and other HIV proteins. It is believed that the transportation of PIC requires the action of IN [166], for the integration of viral cDNA into the host cell genome, an important step in HIV infection [167]. The inhibitory activity of IVM on IN was tested and compared with mifepristone (a standard drug that works by inhibiting IN) using HeLa cells infected with VSV-G-pseudotyped NL4-3.Luc.R-E-HIV. IVM at 25 µM significantly inhibits the growth of the virus. The inhibition of importin this is consistent with ivermectin being able to generally inhibit Impα/β1-mediated nuclear import, which is essential for HIV infection and the first demonstration that inhibitors of nuclear import can have potent antiviral activity. DENV NS5 protein is critical in the viral replication of DENV in the host cytoplasm. Most DENV Ns5 proteins are found in the nucleus at a specific stage of the life cycle of viral infection [154]. NS5 contains another NLS recognized by Impβ1 alone that is not important for the accumulation of NS5nuclear [154]. It was concluded that IVM significantly inhibits the binding of Impα/β1 to NS5 and not the binding of Impβ1 to NS5, showing that the inhibitory activity of IVM was specific, a situation not observed with mifepristone. With respect to dengue virus (DENV) infection, IVM was also studied on its antiviral status against DENV [153,168]. Ivermectin can block the DENV 2 at any anatomical barrier, like midgut or salivary gland the transmission rate of the virus by a mosquito is usually lower than the infection. It is believed that the transmission rate is low as it involves several anatomical barriers such as the midgut and glands. Thus, research is often conducted on the effect of IVM on infection status rather than transmission [169]. They find out that IVM significantly reduced the DENV infection when the vector is pretreated with IVM. IVM could work either in preventing the transport of DENV to the salivary gland or reducing the number of mosquitoes that carry the virus [43]. This means that IVM could be a potential drug used to control DENV infection by acting as an insecticidal [170] in killing *A. albopictus* [171], or as an antiviral drug for the treatment of dengue [169]. IVM prevents the growth of the virus in *Aedes albopictus* as well as destroys several blood-sucking vectors. More research are still been done to decipher the mechanism involved in how IVM affects the transmission and infection rate of DEN through *Aedes albopictus*. One of the mechanisms that IVM might be probably linked to inhibiting the growth of DENV might be by preventing the interaction of NS5 and Impα/β1 [152]. On the basis of the available structures of DENV bound to ssRNA (PDB 2JLU) it is not possible to predict a plausible interaction site or a model of the ternary complex. Reasonably, during activity the helicase/RNA complex changes its structure [172], allowing ivermectin to interact with the identified amino acids to block dsRNA unwinding. IVM has also been reported to be effective against another flavivirus, the yellow fever virus (YFV) [173,174].

### 6.11. Ivermectin and Coronavirus

With the global pandemic nature of COVID-19, caused by severe acute respiratory syndrome coronavirus-2 (SARS-COV-2), there is an urgency for scientists to discover drugs or compounds to treat the disease. The reported antiviral potential of IVM has made it a potential drug to be investigated for the treatment of COVID-19 [175]. Caly et al. [176] earlier reported the potential of IVM to inhibit the replication of SARS-CoV-2 in an in vitro experiment. Such findings were obtained by incubating 2.19 mg/mL of IVM with a Vero/hSLAM-cells infected with SAES-CoV-2. Extrapolating this dose into human models shows that this dose well exceeds tolerable dose for humans approved by the US food and Drug administration. However, Guzzo et al. [131] showed that administration of 120 mg of IVM to healthy humans was safe and well tolerated by volunteers. The results showed the peak plasma concentration of 250 ng/mL, which was just a bit lower than the effective concentration of IVM that inhibits replication of SARS-COV-2 in Vero/hSLAM cells. The results might have been a setback in further clinical trial of IVM. With the respiratory destructive nature of SARS-CoV-2 and Lespine et al. [177] showing a higher concentration of IVM in the respiratory system as compared to the plasma, one week after administration of IVM, shows that all hope might not be lost in the investigation of IVM as a potential antiviral drug. However, some care must be considered before the continuity of the investigation. Based on the reported neurotoxicity and metabolic pathway of IVM, caution should be taken to conduct clinical trial on its antiviral potentials. The GABA-gated chloride channels in the human nervous system might be a target for IVM, this is because the BBB in disease-patient might be a weakened as a result of inflammation and other destructive processes, allowing IVM to cross the BBB and gain access to the CNS where it can elicit its neurotoxic effect (II) IVM is metabolized by CYP3A4 enzyme that is repeatedly inhibited by ritonavir and cubocistat, two drugs used to treat COVID-19 patients. Furthermore, the two drugs are also reported to impair BBB. Thus care should be taken in the administration of IVM with antiviral drugs [178]. Data obtained from the WHO-supported International Drug Monitoring Program are essential in the combination therapy of IVM with other antiviral drugs. They reported that out of 1688 data on co-administration of IVM as an antiviral drugs, only one is linked to its neurotoxicity. (III). the third point is based on the evidenced stated earlier that the potency of IVM to treat SARS-CoV-2 infection might require a dosage that might be toxic to humans [178].

### 6.12. Metabolic Effects of Ivermectin

The role of IVM in regulating lipid and glucose level was investigated using a high-fat diet animal of two types- FXR wild type and FXR −/− mice. Animals were administered IVM intraperitoneally daily for 14 days and concomitantly exposed to a high-fat diet. All the markers of liver injury (aspartate aminotransferase and alanine transferase) along with tissue histology showed no toxicity of IVM [179]. IVM significantly reduced the serum level of cholesterol, low-density cholesterol, very low-density cholesterol, and glucose only in FXR-wild type mice without any change in appetite and weight gained. It is reported that IVM-induced glucose decrease might be due to increased insulin sensitivity to glucose [122]. The role of IVM as an FXR ligand in metabolic regulations was further confirmed by its ability to regulate the expression of some genes that are targeted either directly or indirectly by FXR. mRNA that codes for the expression of SHP, CYP7A1 and CYP8B1. IVM suppresses the expression of CYP7A1, and CYP8B1 mRNA but induces the expression of SHP mRNA. IVM also regulates some key regulatory enzymes in glucose synthesis such as G6Pase and PEPCK. However IVM was only active in FXR wild mice when it was not effective in FXR−/− mice. In this mice (FXR wild type), IVM suppresses the expression of genes involved in G6Pase and PEPCK synthesis, leading to their low concentration [180]. This also confirms the importance FXR in the metabolic activity of IVM. Duran-Sandoval et al. [181] also showed that IVM does not have any effect on the expression of L-PK mRNA levels as compared to GW4064 in hepatic cells and tissues. Apart from regulating genes involved in glucose metabolism, IVM also affects genes involves in cholesterol metabolism. Important regulatory enzymes, such as mRNA that codes for hydroxymethylglutaryl CoA reductase and hydroxymethylglutaryl Coenzyme A synthase, are suppressed by IVM in an FXR-dependent pathway [182]. In addition to these regulatory enzymes, IVM also induced the expression of mRNA that codes multidrug resistance genes, bile salt excretory pumps, and type I scavenger receptor. In another study, IVM caused a significant reduction in the serum cholesterol levels when administered to animal fed high-fat diet and a decrease in body weight was also observed despite the fact that IVM did not influence the animal feed intake rate. IVM also regulates the level of glucose and insulin levels. Their results also corroborated the reported hyperglycaemic and hyperlipidemic effects of IVM. In comparison to the above result, Jin et al. [122] using diabetic mice fed with a high-fat diet, showed that IVM had no significant effect on some regulatory enzymes involved in cholesterol metabolism. That is the mRNA gene that codes for FDFT1 (farnesyl-diphosphate farnesyltransferase 1), hydroxymethylglutaryl coenzyme A synthase, LDL receptor, and hydroxymethylglutaryl coenzyme A reductase were not affected. However IVM alters the mRNA expression of key glucose synthesis enzymes (G6Pase and PEPCK). The summary of pharmacological activities of avermectins are shown in Table 3.

## 7. Pharmacokinetics

There are several factors that determine the pharmacokinetics of ivermectin. Some of the factors include species, weight, body physiology, nutrition, vehicles used in drug preparation and administration route in the animals [183]. However, the administration route and vehicle used in drug preparation are the major factors determining the bioavailability and half-life of the drug [184]. Finally, ivermectin (150 μg/kg) was administered to 16 individuals with water or orange juice (750 mL). Chen et al. [185] reported that orange juice reduced the area under the curve (AUC) of 15.7 ng/d/mL and average maximum plasma concentration (*C*_max_) of 20.7 ng/mL (water: 33.8 ng/mL; 24.3 ng/d/mL), likely because fruit juices and components are potent inhibitors of other drug carriers. The pharmacokinetics (PK) of orally administered ivermectin was tested in 12 healthy male volunteers between 18 and 50 years. A single dose of 12 mg/kg as a tablet resulted in an average maximum plasma concentration peak time of (*T*_max_) 3.6 h, an average maximum plasma concentration of 46 ng/mL and C_max_ of 880 ng/h/mL without showing any clinical side effects [186]. Moreover, Schulz et al. [187] documented that administration of ascending doses of ivermectin resulted in an increase in *C*_max_ and AUC of 23 ng/mL and 350 ng·h/mL in pre-school-aged children (PSAC) and school-aged children (SAC), respectively. They found that PSAC with lower BMI was correlated with substantially higher AUCs and these findings were two-times lower in children relative to previously studied parameters in adults. For PSAC treated with 100 and 200 μg/kg ivermectin, *C*_max_ increased with median values of 15.5 and 24.4 ng/mL, respectively, while for SAC treated with 200, 400 and 600 μg/kg ivermectin, *C*_max_ increased with 21.9, 40.7 and 66.1 ng/mL, respectively. In addition, AUC_0–72_ increased with ascending doses from 331 to 880 to 1636 ng·h/mL in SAC and from 169 to 369 ng·h/mL in PSAC treated groups. Half-life (*t*_1/2_), *T*_max_, V/F and mean residence time (MRT_INF_) values of 18 h, 6 h, 8 L/kg, and 28 h, respectively were similar in all treated groups and therefore independent in dose and age [187]. Schulz et al. [188] reported that *C*_max_ of ivermectin was 51.6 and 40.1 ng/mL for plasma and dried blood spot samples, respectively and observed a slow clearance and a long *t*_1/2_ (26 and 32 h, respectively) in plasma and dried blood spot samples. AUC values were 987 and 810 ng/mL/h for plasma and dried blood spot samples. Ivermectin is well circulated within the blood, where it binds plasma protein, the largest amount is found in the liver, while the lowest amount is found in the brain due to restriction by the blood-brain barrier. It is metabolized by the cytochrome P450 system in the liver, and excreted almost entirely in feces [189]. The uneven distribution among different organs is a major factor determining the varying toxicity of ivermectin between invertebrates and their mammalian hosts. Recent studies to determine the concentration of the drugs that bind to nerve tissues isolated from insects, nematodes, and rat brain, propose that avermectin can elicit its effect at a picomolar or nanomolar concentrations. Using free-living *C. elegans* that is sensitive to avermectin, have been identified as a binding site specific to avermectin from its crude membrane preparation having a dissociation constant (Kd) of 0.26 nM, the Kd for rat brain was found to be 22 nM, and a 100 fold decrease affinity for avermectin. Avermectin is easily absorbed and metabolized by the liver and 98% in the excreta and 1% in the urine, when administered orally on an empty stomach [190]. A trace amount of the drug has been noticed in human milk. After 4 h of ivermectin administration, the drug concentration peaks at 30–46 ng/mL in the blood, which slowly decreases with time. [186,191]. The concentration of metabolites increases in the blood for a longer period of time compared to the parent compound, which may be due to heterohepatic recycling. Ivermectin has been identified in the nodules, skin fat, and subcutaneous fascia [192]. A single 12 mg oral administration of the drug, peaks at 8 h in the antihenar, sebum, squames, and forehead and begin to reduce after 24 h [183].

## 8. Environmental Effects of the Usage of Avermectins in Livestock

Abamectin (avermectin B1) and ivermectin (22, 23-dihydroavermectin B1) are high molecular weight hydrophobic compounds that are active against a variety of animal parasites and insects [193]. Numerous environmental fate and effects studies have been carried out in the development of these two compounds as antiparasitic agents and for abamectin as a crop protection chemical. Two essential AVM drugs, abamectin (avermectin B1) and ivermectin (22, 23-dihydroavermectin B1), have been subjected to numerous environmental studies as they are widely used for crop protection and pest control. Their high molecular weight makes them immobile in soil (*K*oc > or4000), easily degraded in soil under aerobic conditions, *t*_1/2_ of 2–8 weeks for abamectin in soil and 7–14 days for IVM in soil/faeces, in water the (*t*_1/2_) for degradation was less than 12 h and less than 24 h as thin films on surfaces to less bioactive compounds. Plants cannot absorb abamectin from the soil, nor is it bio accumulated in fish. A study showed that Daphnia magna is the most sensitive water species to abamectin and IVM with an LC_50_ values of 0.34 ppb and 0.025 ppb, respectively, while fishes are the least sensitive with LC_50_ values of 3.2 ppb and 3.0 ppb, respectively. Sediments and other particles present in water that can degrade AVM and abamectin often reduce their toxicity in Daphnia. They have a little toxic effect on avian, for example, abamectin has an LC_50_ values of 3102 ppm and 383 ppm for bobwhite quail and mallard duck, respectively, while in the earthworm, IVM and abamectin has LC_50_ value of 315 ppm and 28 ppm, respectively. Residues of avermectin in livestock feces have been reported to affect the larval form of some dung-associated insects. However, usage pattern of the drugs can limit their effects on dung-associated insects depending on the availability of dung without the drugs and mobility of the insects. The above properties of IVM and abamectin concerning biodegradation often limit their bioaccumulation and translocation in the environment and minimize exposure to non-target organisms or location.

## 9. Clinical Trials of Avermectins

Over 20 years, ivermectin has been used for in the treatment of human onchocerciasis and lymphatic filariasis [75]. Recent studies concerning ivermectin warrant the swift implementation of controlled clinical trials to evaluate its effectiveness against SARS-CoV-2 that can open up a new field of study on the prospective use of avermectin [178]. Moreover, a recent stage III clinical trial in dengue patients in Thailand showed that daily administration of 400 μg/kg for 3 days showed a modest effect against dengue virus [194]. Another clinical trial was conducted at six health facilities in western Kenya and requires a sample size of 141 participants to detect the pharmacokinetics, efficacy and safety of ivermectin to provide a promising new malaria elimination tool [195]. Three in vivo studies evaluated the long-term effect of ivermectin on the survival of mosquitoes by feeding at least seven days after ivermectin administration [196,197,198]. Chaccour et al. [196] revealed that the administration of single low dose of 200 µg/kg ivermectin increased mosquito mortality by 1.33-fold when they fed on blood taken from humans received ivermectin one day earlier, however, no effect was observed when mosquitoes were fed on blood taken on day 14 post-treatment. Bastiaens et al. [198] recorded that a repeated dose of 200 μg/kg administered on days zero and two had a modest effect on decreased survival seven days after treatment, while Bryan et al. [197] reported that administering 250 µg/kg to a single human volunteer had a significant impact for at least two weeks after treatment. Other avermectins such as moxidectin, selamectin, and doramectin, used to control nematodes in livestock and pets in veterinary medicine are identified to be safe and well-accepted [75]. A Phase III study was conducted to compare the efficacy, tolerability and safety of 2, 4, and 8 mg of moxidectin and ivermectin oral administration in individuals with *Onchocerca volvulus* infection, the parasite causing river blindness. The 8 mg dose of moxidectin was found to be safe to human, confirming its ability to monitor onchocerciasis elimination [199]. Selamectin can be administered orally, topically, or subcutaneously in the veterinary medicine for the treatment of various ecto- and endoparasites in cats and dogs, but is not currently approved for human use [200]. The summary the current clinical trials/human on avermectins are shown in Table 4.

## 10. Toxicity and Side Effects

As with most drugs, avermectin also has unwanted effects or reactions, especially when administered indiscriminately [201]. The toxicity of different types of AVM has been reported either singly or in combinations [166]. Some of the biochemical processes reported to be affected by AVM includes blocking of lipopolysaccharide (LPS) induced release of tumor necrosis factor (TNF), prostaglandin E2 (PGE2), nitric oxide (NO), and the increase in the concentration of intracellular calcium ion (Ca^2+^) [202]. Severe effects of AVM are often related to overdose intake, thus it is not a common experience and includes respiratory failure, hypotension, and coma. Unless it is not treated rapidly, it can lead to death. Though no specific treatment is available, the observed symptoms can be managed until condition stabilized [203]. The mechanism of AVM by which they elicit their pharmacology and toxicology is similar, despite the differences in their structure, potency, and safety. Such mechanisms also involved the glutamate-gated chloride channels and or GABA receptors in the nerve and muscle cells in the invertebrate, resulting in their paralysis and death. The intoxication process of AVM often begins with hyper excitability, uncontrolled, and uncoordinated movement of the muscle and body. This then leads to ataxia and immobility like-coma. The enhanced permeability of chloride ion across the membrane and GABAA agonist’s mimicking-effect of AVM as stated earlier can add to the toxicity of AVM. The toxicity of AVM is more pronounced in animals with a compromised p-glycoprotein. Their results also showed that IVM is not genotoxic as various studies using different models to screen for defects in DNA synthesis, clastogenicity, and mutagenicity. The first AVM to be released for the treatment of animal and human parasitic infections was ivermectin, based on its high efficacy and tolerability. It was later followed by the approval and commercialization of others, such as abamectin, emamectin, and moxidectin, as an acaricides or insecticides and parasiticides for crop protection and animal health, respectively. It was reported that the toxicity of IVM use is rare and most side effects are often transient and mild to moderate. Most observed symptoms are generally treated by available measures. Some observable toxicity may also be caused by the use of avermectin for non-labelled purposes. However, overdose or uncontrolled administration can lead to the development of severe toxicity. To illustrate the role of species differences in the expression of efflux transporter, Stevens et al. [204] investigated the effect of the placenta-fetal barrier on the toxic effect of AVM using rodents. They concluded that the use of animal models in toxicological research and extrapolation to humans is necessary to note the variation in genetic makeup between organisms and between animals and humans.

### 10.1. Neurotoxicology of Ivermectin

The major toxic effects of IVM observed in the animal studies using rats are ataxia, sluggishness, and ptosis, while in dogs, hypersalivation, ataxia, blindness, coma, respiratory compromise, tremors, mydriasis, anorexia, and death [3]. It was also noted that young rats are sensitive to IVM, as a high concentrations of IVM are found in the brain due to a tender BBB and lack of p-glycoprotein. Most dogs sensitive to IVM neurotoxicity are due to a mutation in MDR1 gene as a result of 4 –bp deletion. Mutation of this gene leads to an incomplete synthesis of p-glycoprotein [205]. The role of P-glycoprotein (P-gp) is critical in the potential neurotoxicity of IVM. P-gp is part of the important component of the BBB and it is important in the process by which the BBB regulates the entry and exit of drugs into the brain. Therefore, any activity that regulates the function of P-gp can affect the concentration of IVM in the brain, especially the efflux of IVM from the brain. Some of the processes that affect the function of P-gp include mutation and administration of drugs that inhibit its ability to transport drugs out of the brain. Some groups of mice that have a low levels of p-glycoprotein and another group that does not express p-glycoprotein, indicated with (−/−) [206]. The sensitivity of animals with deficient genotype or wild type (+/+) to neurotoxicity or teratotoxicity of IVM also depends on its concentration in the brain and fetus. It was showed that mice deficient in p-glycoprotein share the same phenotype with MDR1a and MDR1b knockout mice [143]. These knockout mice were used to prove that the accumulation of IVM in the brain was due to differences in the tissue distribution and pharmacokinetics of IVM as compared to wild type mice [207]. To further establish the role of MDR1a transporter in bioavailability and brain uptake of IVM, cyclosporine, a substrate for p-glycoprotein and IVM absorption were enhanced in the (−/−) CF-1 mice strain without affecting the intravenous pharmacokinetics of either drugs [208]. This shows that metabolism of the drugs in the liver between the two strains ((−/−) and (+/+)) of animals is similar, whereas the concentration of cyclosporine A and ivermectin in the brain has increased relative to the wild form (+/+) of mice independent of oral or intravenous administration. This confirms that changes and sensitivity of animals to IVM is as a result of p-glycoprotein deficiency, not adjustment in drug metabolism [208]. HIV protease inhibitors are another drug that can increase the toxicity of IVM in the brain. HIV protease inhibitors are a substrate for P-gp and might compete with IVM for its binding, leading to accumulation of IVM in the brain that might further increase its toxicity in the brain. Both albendazole and IVM are metabolized by CYP3A4, a drug metabolizing enzymes that is abundant in the liver and small intestine [209]. With most studies conducted so far showing that albendazole is not a substrate for P-gp, it can be concluded that the effect of albendazole on the increased toxicity of IVM might be connected to its clearance from the liver [210]. In finding an answer to whether IVM neurotoxicity is due to drug metabolizing enzymes or P-gp, Nobmann et al. [211] used an IVM labelled with a fluorescent compound (BODIPY) tagged BODIPY-labelled IVM. They found out that the efflux of the drug is reduced by a substrate that binds to P-gp. Further investigation showed that the dosage required for drug-drug interaction to be effective as a co-substrate for P-gp might not be possible [212]. Based on the inability of some antipsychotic, antiemetic and Ca^2+^ blocking agents could not replicate their inhibitory activity of P-gp as achieved in the systemic circulation using suitable concentrations.

### 10.2. Toxic Effect of Ivermectin

Ivermectin, the most widely used AVM has been reported to be widely safe by several authors, they concluded that most mammalian species respond very well to IVM exposure. However, few animals show high sensitivity to IVM toxicity. Chhaiya et al. [190] reported that some patients with filarial infection developed transient to mild side effects when administered IVM. Anorexia, headache, myalgia, arthralgia, fever, eosinophilia, and asthenia are some of the symptoms reported. Metabolic products of the microfilaria released in filarial patients has been reported to cause Mazzotti reactions and sudden death. Also, some scabies patient administered IVM developed popular rashes and pruritus (33%) 2 to 4 days later. Other symptoms observed in some of the patients administered IVM include hematomas, prolonged in blood prothrombin time, nausea, decrease in blood pressure, and flat T waves on ECG [213]. Neonatal mice, which have an immature blood brain barrier, and adult mice and dogs with defective P-glycoprotein are most at risk of ivermectin toxicity, even when given at doses as low as 0.4 mg/kg [214,215,216]. In addition, typical therapeutic doses have been shown to cause adverse effects in various mouse strains or stocks, and dog breeds with normal levels of P-glycoprotein [217]. Also, ivermectin has been shown to alter aspects of behavior and immune function in mice to indicate that the immunostimulatory properties of ivermectin are associated with altered function of T lymphocytes, in particular, T-helper lymphocytes. The immunomodulating effects of ivermectin may provide an alternative approach for the treatment of disease problems involving immunosuppression [218,219]. Davis et al. [220] also find out that IVM has no significant effect on most parameters evaluated when used to treat mite-infected mice within a barrier facility. Molinari et al. [221] concluded that while IVM and ABM did not cause gene mutations in various in vitro and in vivo models using bacteria or animal cell. Neither was there any significant proof of clastogenic effect induced on animal cells. The scarce information on claims of the drugs causing breaks in single strands DNA or inhibit cell growth either in an in vitro or in vivo bioassays. The claimed genotoxicity and cytotoxicity of the two drugs needs to be thoroughly investigated and evaluated for the hazard classification, especially ABM, which is classified as a class II toxicity pesticides by EPA. Previously published toxicology testing of ivermectin yielded conflicting results. One study reported daily exposure of adult CD1 mice to doses as high as 12 mg/kg for as long as 94 day resulted in only a 29% decreased body weight and no other clinical signs [215]. In another study, doses as low as 0.2 mg/kg resulted in mortality, convulsion, and coma in pregnant female mice and 0.4 mg/kg caused cleft palate in pups exposed in utero [222]. This finding established a no-effect level in mice at 0.1 mg/kg and the belief that mice are uniquely sensitive to ivermectin toxicity. As a comparison, no-effect levels in rats at 5 mg/kg [215]. However, oral ivermectin at doses as high as 2.0 mg/kg has been used to treat mice without adverse effects [223]. Most published treatment regimens for mice range from 1 to 1.9 mg/kg all of which are clearly above the 0.1-mg/kg no effect level cited [224,225,226,227,228]. To maximize the probability of successfully eliminating fur mites from their large mouse colony, they used the highest dose tested that lacked adverse effects in their toxicity study. Because most colonies in their vivarium are maintained on a C57BL/6 (B6) background, toxicity experiments were conducted on mice of this strain. They found that adult B6 mice were relatively resistant to high doses of ivermectin; only 1 of 144 mice receiving 5.4 mg/kg ivermectin daily (48 ppm diet) demonstrated clinical signs. Although gross necropsy and histologic evaluation of tissues from this mouse were unremarkable, the nature of the clinical presentation (tremors, ataxia) and the timing (2 d after initiation of treatment) are suggestive of ivermectin toxicity. Most reports do not describe mortality at doses equivalent to 12- and 24-ppm feed (1.3 and 2.7 mg/kg), but one study reported that 2 of 22 129/SvSj mice died after receiving a dose of 1 to 2 mg/kg [229]. Pathology was not performed and clinical signs were not observed in the cited study; it is possible that the mice died of unrelated causes, especially because an additional 10 129/SvSj mice receiving 5 times the original dose did not demonstrate toxicity [230]. Furthermore, 129/SvEv mice treated with oral ivermectin in water for 8 weeks at doses between 1.2 and 1.9 mg/kg demonstrated no adverse effects [231]. In their study, body weight gain among the P0 generation remained constant through the first 3 weeks of treatment in all groups [232]. Nine days after the cessation of treatment (day 65), mice receiving 5.4 mg/kg (48 ppm diet) ivermectin were smaller than control mice. Unfortunately, the day 43 weight for the 48-ppm group was not recorded, so these animals may have gained less weight during the second half of the treatment period than during the first half; however, at day 181, no weight differences were observed between groups. Weight loss was the only adverse finding reported in the initial toxicology study of ivermectin in adult CD1 mice [214]. Even though the dose used in the cited study (12 mg/kg) was more than two-fold greater than the highest dose evaluated (5.4 mg/kg), their findings are consistent. Comparing body weight at 32 weeks of age among mice in the P0, F1 (control diet), and F2 (control and 12-ppm diets) generations revealed that F1 and F2 mice were significantly larger than their P0 counterparts. This result may reflect the feed these animals had received. The P0 generation was obtained from Taconic Farms at six to eight weeks of age and had been fed NIH no. 31M Rodent Diet prior to their arrival. This diet has a considerably lower fat content (5.3%) than does the Purina 5058 feed (9% crude fat content) used [233]. However, weight gain was not observed in the group that received 2.7 mg/kg ivermectin. There was no weight increase over subsequent generations, and the treated F1 mice were significantly smaller than those in the control F1 group. Exposure to ivermectin as neonates apparently can reduce the animals’ adult weight, even after drug exposure had ceased. Interestingly, the average weaning weight of F1 mice born during ivermectin treatment was very similar between dosage groups and to the weaning weight of animals born after the end of treatment. During the preweaning period, all pups gained weight at the same rate regardless of ivermectin exposure. The preweaning growth rate most likely reflects the dam’s milk production. Despite identical weaning weights, the ability of these animals to gain weight throughout their adult lives may be impaired by neonatal ivermectin exposure. The main adverse effects of ivermectin in the original toxicology study were seen in pregnant dams, embryos, and neonatal pups [215]. None of the pregnant mice showed any clinical signs or mortality in any of the treatment groups. In addition, they also observed no teratogenic effects (for example, cleft palate) in any of the pups that had been exposed to ivermectin at any dose in utero. Their results also showed no differences in the numbers of litters or pups born to dams of the various treatment groups. In summary, their findings indicate that ivermectin at doses as high as 5.4 mg/kg does not affect the fertility of C57BL/6NTac mice. Of importance, dose-dependent increases in the mortality rates were noticed in neonatal mice in the 2.7- and 5.4-mg/kg groups. In the highest dose group, none of the pups survived past the fourth day. This effect was transient, and litters born in the 12- and 48-ppm groups after cessation of treatment had identical survival rates. Even pups born immediately after treatment ended and that therefore had been exposed to ivermectin in utero for most of the pregnancy survived. This finding indicates no measurable intrauterine effect of ivermectin on the pups. The increased mortality in neonates in the higher dose groups is most likely due to two processes. Nursing pups are exposed to ivermectin through milk. Because ivermectin is lipophilic, it concentrates in the milk, and the pups’ oral exposure level is higher than that of the dam. In addition, the blood–brain barrier of rodents is not fully developed until approximately 10 d of age. One study reports similar findings in rats exposed to ivermectin and attributes neonatal mortality to the accumulation of ivermectin to toxic levels in the brain [234]. Although adult toxicity was studied in CD1 mice, reproductive toxicity was studied in CF1 mice. The CF1 stock later was found to have a high percentage of mice with a P-glycoprotein deficiency due to a natural mutation [214]. This deficiency markedly increases the susceptibility of these mice to ivermectin toxicity. The unfortunate use of this highly susceptible stock in the toxicity study gave rise to the idea that mice in general are highly susceptible to ivermectin toxicity. Ricart et al. [232] did not observe any adverse effects of ivermectin exposure on the fertility or fecundity of offspring of C57BL/6NTac mice treated with ivermectin. In conclusion, their results suggest that ivermectin can be compounded in rodent feed and, although a decline in drug concentration occurs due to manufacturing, the remaining drug is stable for at least six months post-milling. Ivermectin can be safely administered to neonatal, juvenile, adult, and pregnant C57BL/6NTac mice at 1.3 mg/kg daily for an 8-wk period. Further studies should be conducted to assess safety in other strains of mice, including those known to be highly susceptible to ivermectin toxicity. Pregnant mice in their studies did not show any clinical signs, teratogenic effects, differences in the numbers of litters or pups born to dams of the various treatment groups or mortality in any of the treatment groups [232]. Together, these findings indicate that ivermectin at doses as high as 5.4 mg/kg has no effect on the fertility of C57BL/6NTac mice.

## 11. Conclusions

This review examines the medicinal and side effects of avermectins. Avermectins are a remarkable compounds which were first isolated in 1978. Avermectins are generally soluble in organic solvents, such as ethanol, chloroform, diethyl ether, ethyl acetate and have a high adsorption coefficient, which makes them less likely to accumulate in the water column. They are commonly used in oral, parenteral, topical, or spot topical (as in veterinary flea repellant “drops”) form and show activity against a broad range of nematodes and arthropod parasites of domestic animals at doses of 300 μg/kg or less than 200 μg/kg. Ivermectin, the most widely used AVM has been reported to be widely safe for most mammalian species. Several reports documented that administration of ascending doses of ivermectin resulted in an increase in *C*_max_, *T*_max_, *t*_1/2_ and AUC in tested volunteers. Ivermectin is well circulated within the blood, where it binds plasma protein, the largest amount is found in the liver, while the lowest amount is found in the brain due to restriction by the blood-brain barrier. It is metabolized by the cytochrome P450 system in the liver, and excreted almost entirely in feces. IVM and abamectin concerned biodegradation that often limits their bioaccumulation and translocation in the environment and minimized their exposure to non-target organisms or location. Over 20 years, ivermectin has been used for in the treatment of human onchocerciasis and lymphatic filariasis. Recent studies concerning ivermectin warrant the swift implementation of controlled clinical trials to evaluate its effectiveness against SARS-CoV-2 that can open up a new field of study on the prospective use of avermectin. The major toxic effects of IVM observed in the animal studies using rats are ataxia, sluggishness, and ptosis, while in dogs, hypersalivation, ataxia, blindness, coma, respiratory distress, tremors, mydriasis, anorexia, and death.

## Figures and Tables

**Table 1:** The chemical structures, synonyms, IUPAC name, and molecular formula of ivermectin, selamectin, moxidectin, eprinomectin, nemadectin, abamectin, milbemycin and doramectin.

Compound	Chemical Structures	Synonyms	IUPAC Name	Molecular Formula	Ref.
Ivermectin	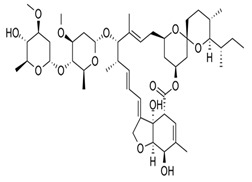	Eqvalan Ivermectin Ivomec Mectizan MK 933 Stromectol	1*R*,4*S*,5′*S*,6*R*,6′*R*,8*R*,10*E*,12*S*,13*S*,14*E*,16*E*,20*R*,21*R*,24*S*)-6′-[(2*S*)-butan-2-yl]-21,24-dihydroxy-12-[(2*R*,4*S*,5*S*,6*S*)-5-[(2*S*,4*S*,5*S*,6*S*)-5-hydroxy-4-methoxy-6-methyloxan-2-yl]oxy-4-methoxy-6-methyloxan-2-yl]oxy-5′,11,13,22-tetramethylspiro[3,7,19-trioxatetracyclo[15.6.1.1^4,8^.0^20,24^]pentacosa-10,14,16,22-tetraene-6,2′-oxane]-2-one	C_48_H_74_O_14_	[39]
Selamectin	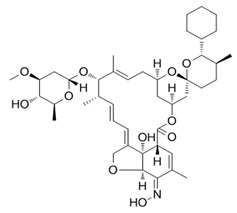	Selamectin UNII- 220119-17-5 A2669OWX9N UK-124,114	((2a*E*,4*E*,8*E*,20*Z*)-(5′*S*,6*S*,6′*S*,7*S*,11*R*,13*R*,15*S*,17a*R*,20a*R*,20b*S*)-6′-cyclohexyl-3′,4′,5′,6,6′,7,10,11,14,15,17a,20,20a,20b-tetradecahydro-20b-hydroxy-20-(hydroxyimino)-5′,6,8,19-tetramethyl-17-oxospiro[11,15-methano-2*H*,13*H*,17*H*-furo[4,3,2-*pq*][2,6]benzodioxacyclooctadecin-13,2′-[2*H*]pyran]-7-yl 2,6-dideoxy-3-*O*-methyl-α-L-*arabino*-hexopyranoside	C_43_H_63_NO_11_	
Moxidectin	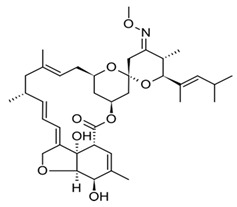	ProHeart 6 CL301423 Cydectin	1*R*,4*S*,5′*S*,6*R*,6′*S*,8*R*,10*E*,12*S*,13*S*,14*E*,16*E*,20*R*,21*Z*,24*S*)-6′-cyclohexyl-24-hydroxy-21-hydroxyimino-12-[(2*R*,4*S*,5*S*,6*S*)-5-hydroxy-4-methoxy-6-methyloxan-2-yl]oxy-5′,11,13,22-tetramethylspiro[3,7,19-trioxatetracyclo[15.6.1.1^4,8^.0^20,24^]pentacosa-10,14,16,22-tetraene-6,2′-oxane]-2-one	C_37_H_53_NO_8_	
Doramectin	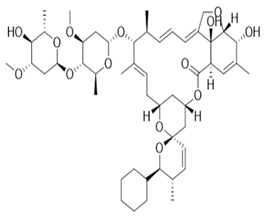	DECTOMAX UK-67,994	(1′*R*,2*R*,3*S*,4′*S*,6*S*,8′*R*,10′*E*,12′*S*,13′*S*,14′*E*,16′*E*,20′*R*,21′*R*,24′*S*)-2-cyclohexyl-21′,24′-dihydroxy-12′-[(2*R*,4*S*,5*S*,6*S*)-5-[(2*S*,4*S*,5*S*,6*S*)-5-hydroxy-4-methoxy-6-methyloxan-2-yl]oxy-4-methoxy-6-methyloxan-2-yl]oxy-3,11′,13′,22′-tetramethylspiro[2,3-dihydropyran-6,6′-3,7,19-trioxatetracyclo[15.6.1.1^4,8^.0^20,24^]pentacosa-10,14,16,22-tetraene]-2′-one	C_50_H_74_O_14_	
Eprinomectin	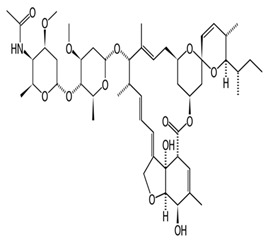	Avermectin B1, 4″-(acetylamino)-4″-deoxy-, (4″R)- Eprinex Eprinomectin [USAN:USP:INN] MK 397 ZINC306122586	(*N*-[(2*S*,3*R*,4*S*,6*S*)-6-[(2*S*,3*S*,4*S*,6*R*)-6-[(1′*R*,2*R*,3*S*,4′*S*,6*S*,8′*R*,10′*E*,12′*S*,13′*S*,14′*E*,16′*E*,20′*R*,21′*R*,24′*S*)-21′,24′-dihydroxy-3,11′,13′,22′-tetramethyl-2′-oxo-2-propan-2-ylspiro[2,3-dihydropyran-6,6′-3,7,19-trioxatetracyclo[15.6.1.1^4,8^.0^20,24^]pentacosa-10,14,16,22-tetraene]-12′-yl]oxy-4-methoxy-2-methyloxan-3-yl]oxy-4-methoxy-2-methyloxan-3-yl]acetamido	C_49_H_73_NO_14_	
Nemadectin	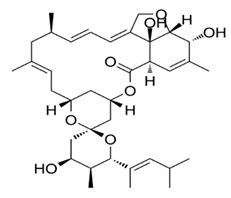	Nemadectin UNII-1Y8VJ1G3TY 1Y8VJ1G3TY 102130-84-7 Nemadectina	((1*R*,4*S*,4′*S*,5′*S*,6*R*,6′*S*,8*R*,10*E*,13*R*,14*E*,16*E*,20*R*,21*R*,24*S*)-4′,21,24-trihydroxy-5′,11,13,22-tetramethyl-6′-[(*E*)-4-methylpent-2-en-2-yl]spiro[3,7,19-trioxatetracyclo[15.6.1.1^4,8^.0^20,24^]pentacosa-10,14,16,22-tetraene-6,2′-oxane]-2-one	C_36_H_52_O_8_	
Milbemycin	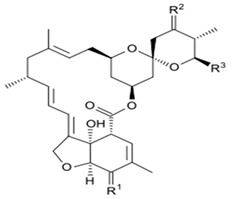	Milbemycin D Antibiotic B 41D Milbemycin B 41D UNII-04S0E2XJQI	((1*R*,4*S*,5′*S*,6*R*,6′*R*,8*R*,10*E*,13*R*,14*E*,16*E*,20*R*,21*R*,24*S*)-21,24-dihydroxy-5′,11,13,22-tetramethyl-6′-propan-2-ylspiro[3,7,19-trioxatetracyclo[15.6.1.1^4,8^.0^20,24^]pentacosa-10,14,16,22-tetraene-6,2′-oxane]-2-one	C_33_H_48_O_7_	
Abamectin	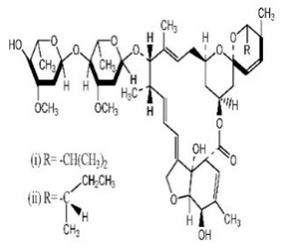	Abamectinum Agrimek Vertimec Affirm Avomec	((1′*R*,2*R*,3*S*,4′*S*,6*S*,8′*R*,10′*E*,12′*S*,13′*S*,14′*E*,16′*E*,20′*R*,21′*R*,24′*S*)-2-butan-2-yl-21′,24′-dihydroxy-12′-[(2*R*,4*S*,5*S*,6*S*)-5-[(2*S*,4*S*,5*S*,6*S*)-5-hydroxy-4-methoxy-6-methyloxan-2-yl]oxy-4-methoxy-6-methyloxan-2-yl]oxy-3,11′,13′,22′-tetramethylspiro[2,3-dihydropyran-6,6′-3,7,19-trioxatetracyclo[15.6.1.1^4,8^.0^20,24^]pentacosa-10,14,16,22-tetraene]-2′-one	C_95_H_142_O_28_	

**Table 2:** Summary of human uses of ivermectin.

Uses	Ref.
*Onchocerca volvulus*	[73]
Strongyloidiasis	[74,75]
Scabies	[78]
*Papulopustular rosacea* (PPR)	[79]
Pediculosis	[74,76]
Gnathostomiasis	
Myiasis	
Leishmaniasis	
Trichuris	
Ascaris	

**Table 3:** Pharmacological activity of avermectin.

Activity	Compound	Organism Active Against	Ref.
Nematocidal	Abamectin	*Hoplolaimus galeatus* and *Tylenchorynchus dubius, E. vermiculata*	[88,89]
Antibacterial	Ivermectin Selamectin Doramectin Moxidectin	*Mycobacterium bovis* and *M. tuberculosis*	[93,96,97]
Anti-plasmodium	Ivermectin	*P. falciparum*	[104]
Anti-inflammatory	Ivermectin	Skin inflammation	[111]
Anticancer	Ivermectin	Lung cancer and colon cancer	[118]
Antiviral	Ivermectin	Dengue virus and yellow fever virus	[28]

**Table 4:** Summary of the current clinical trials/human on avermectins.

Derivative	Clinical Trial	Ref.
Ivermectin	Phase III clinical trial in dengue patient in Thailand shows it is safe but of no clinical benefit	[194]
Moxidectin	Undergoing phase III clinical trial to treat patient *Onchocerca volvulus* infection	[199]
Selamectin	Not approved for human use	[200]
